# Expression of Concern: Fingolimod inhibits proliferation and epithelial–mesenchymal transition in sacral chordoma by inactivating IL-6/STAT3 signaling

**DOI:** 10.1042/BSR-20200221_EOC

**Published:** 2020-06-24

**Keywords:** FTY720, proliferation, epithelial-mesenchymal transition, sacral chordoma, IL-6/STAT3 signaling

The Editorial Office has been made aware of potential issues surrounding the scientific validity of this paper, hence has issued an expression of concern to notify readers whilst the Editorial Office investigates.

